# Factors Conditioning the Potential Effects TiO2 NPs Exposure on Human Microbiota: a Mini-Review

**DOI:** 10.1007/s12011-021-02578-5

**Published:** 2021-01-14

**Authors:** Ewa Baranowska-Wójcik

**Affiliations:** Department of Biotechnology, Microbiology and Human Nutrition, University of Life Sciences in Lublin, Skromna 8, Lublin, Poland

**Keywords:** TiO_2_ NPs, Microbiota, Nanoparticles, Bacterial, Human gastrointestinal microbiota

## Abstract

The recent years have seen a significant interest in the applications of nanotechnology in various facets of our lives. Due to their increasingly widespread use, human exposure to nanoparticles (NPs) is fast becoming unavoidable. Among the wide group of nanoparticles currently employed in industry, titanium dioxide nanoparticles, TiO_2_ NPs, are particularly popular. Due to its white colour, TiO_2_ is widely used as a whitening food additive (E 171). Yet, there have been few studies aimed at determining its direct impact on bacteria, while the available data suggest that TiO_2_ NPs may influence microbiota causing problems such as inflammatory bowel disease, obesity, or immunological disorders. Indeed, there are increasing concerns that its presence may lead to intestinal barrier impairment, including dysbiosis of intestinal microbiota. This article aims to present an overview of studies conducted to date with regard to the impact of TiO_2_ NPs on human microbiota as well as factors that can affect the same. Such information is necessary if we are to conclusively determine the potential toxicity of inorganic nanoparticles.

## Introduction

In recent years, the use of nanomaterials in food products has been observed to grow rapidly on a continuous basis, which inevitably increases the risk of adverse health effects resulting from their uncontrolled release [1, 2].

Numerous in vitro and in vivo studies conducted to date have confirmed the toxicity of TiO_2_ NPs against a human organism, including effects related to cellular cycle alterations, nuclear envelope contraction, and apoptosis [3, 4]. In vivo studies further demonstrated that after inhalation or oral exposure, TiO_2_ NPs accumulate in, e.g. the lungs, heart, alimentary tract, liver, spleen, kidneys, and myocardium, as well as upset the homeostasis of glucose and lipid metabolism in mice and rats [5–7]. Other possible effects include dyspepsia and nutrient absorption disorders after exposure to TiO_2_ NPs, which may be a consequence of micro- and macro-elements in the organism [8]. In the brain, TiO_2_ NPs can trigger protein oxidation, oxidative damage, reduction of antioxidative capacity, and increased production of ROS (reactive oxygen species) [9, 10].

TiO_2_ NPs (nanoparticles) are used as whitening or brightening additive in the food industry (coded—E171). They are commonly added to a number of products including sauces, cheeses, skimmed milk, ice-cream, and confectionary products—e.g. as coating for sweets and chewing gum [11–14]. They are also utilised in food processing and packaging, as well as added to pharmaceuticals, cosmetics, and toothpastes [11, 15, 16]. Owing to their antibacterial properties, TiO_2_ NPs may also serve as food preservatives [17, 18].

TiO_2_ content in confectionary products, particularly in sweets, chewing gum, chocolate, and other white-coated products, can be very high, reaching up to 2.5 mg Ti/g of food [14, 19]. The lack of sufficient research data prevents the determination of the admissible, daily consumption of TiO_2_ NPs [19]. Based on studies conducted on animals, a safety margin of 2.25 mg TiO_2_ NPs/kg bm/day was suggested [19]. Its daily consumption varies depending on age, body weight, and place of residence. It is nonetheless estimated that a child is likely to ingest up to 2–4 times more TiO_2_ NPs per 1 kg of body mass (Table 1) [14, 19, 20] compared to an adult. In Great Britain, children under 10 years old consume, on average, approximately 2–3 mg of TiO_2_/kg bm/day, while in adults this value is estimated at 1 mg TiO_2_/kg bm/day [14].

**Table 1:** Human oral exposure to TiO_2_ NPs in food

Areas	TiO_2_ (mg/kg bw/day)		Reference
	Children	Other ages	
USA	1–2	0.2–0.7	[14]
United Kingdom	2–3	1	[14]
Europe	5.5–10.4	0.2–0.4	[19]
Netherlands	1.4	0.5–0.7	[20]
China (Beijing)	0.02–3.09		[20]

TiO_2_ (mg/kg bw/day)—TiO_2_ NPs per 1 kg of body weight (bw) per day

The impact of TiO_2_ NPs on the human organism has been debated for years. Both the levels of its exposure and toxicity to a human/animal organism have been subject to in-depth study and discussion. The wide-spread use of TiO_2_ NPs in the food industry has raised considerable safety concerns and controversy [11, 21]. Some studies conclude that TiO_2_ NPs may be toxic towards and have adverse effects on the cardiovascular system. Elevated expression of inflammatory cytokines such as TNF-α, INF-g, and IL-8 in the blood, after the ingestion of TiO_2_ NPs, was reported in studies by Gui et al. [22] and Trouiller et al. [23]. When studying the in vivo toxicity of TiO_2_ NPs in mice, Chen et al. [24] observed strong symptoms of toxicity, including loss of appetite, tremors, passive behaviour, or lethargy. Furthermore, in a study on rats, Wang et al. [25] observed hepatic oedema, heart damage, and non-allergic activation of mast cells in stomach tissue. Human organism is strongly dependant on its microbiota in terms of, e.g. the ability to digest dietary fibre and other nutrients, modulation of the host immunological response, food transit in the intestines, and defence against pathogens [26].

Interactions between gastrointestinal microbiota and NPs may affect the host’s health directly, through NPs-induced modification of the microbiota (increased toxicity), or indirectly, due to dysbiosis of gastrointestinal microorganisms [27]. One should also take into account the fact that various interactions between NPs and gastrointestinal bacteria may be dependent of a wide range of factors, e.g. the surface charge of nanoparticles and bacteria, the surface charge of the digested food, the chemical composition of respective substances and diet components [28], as well as the physicochemical conditions inside the alimentary canal (pH, enzymes, salts, etc.) [29].

**Fig. 1:**
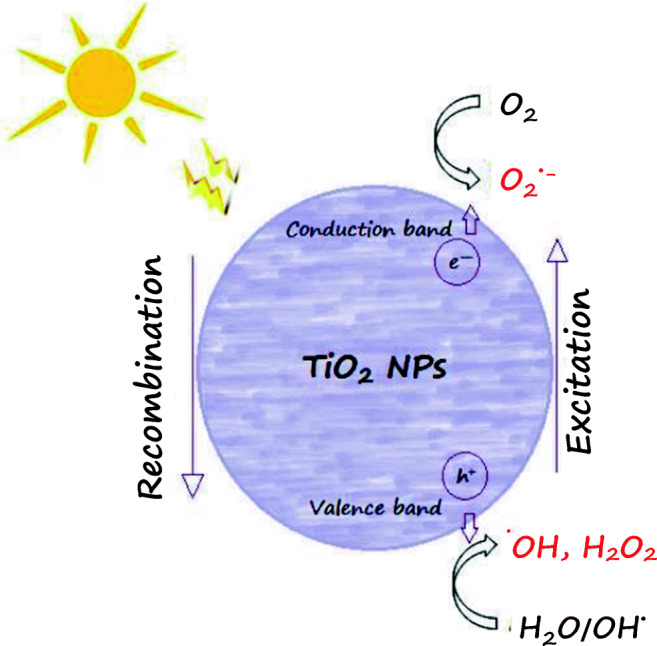
Mechanism of ROS formation on the surface of titanium oxide nanoparticles

As single-cell organisms, bacteria provide a very good test model for analysing the toxicity of nanoparticles, e.g. to determine their impact on the functional health of a cell organism [30]. Nanoparticles interact with bacteria producing reactive oxygen species (ROS), which in turn can damage DNA, RNA, and proteins [31] (Fig. 1). As follows from research, among the TiO_2_NPs, the anatase TiO_2_ forms are more toxic towards bacteria than rutile NPs as they cause greater oxidative stress [32, 33]. As reported by Kim et.al [34], mobile (•) OH is generated in anatase; hence, photocatalytic oxidation therein is easier compared to rutile which can only adsorb a substrate. TiO2 NPs mainly generate electrons and superoxide ions (O2 • -) in the conduction band, as well as positive holes and hydroxyl radicals (• OH) in the valence band. Next, the generated ROS can lead to oxidation of the TiO2 NPs adsorbed on the surfaces of bacteria, leading to their death [35].

Researching the interactions between bacteria and NPs may provide us with a lot of valuable information [30]. There have only been a handful of studies exploring the interactions between NPs and gastrointestinal microbiota, and the resulting impact on the host’s health, with most of the same focusing on the direct interactions with the cells of intestinal epithelium [36, 37], as well as photocatalytic applications in UV light (ultraviolet filter) [38].

This review aims to present detailed results of recent studies pertaining to the effects of TiO2 NPs exposure on human intestinal microbiota, as well as factors that may influence the same.

## Material and Methods

**Fig. 2:**
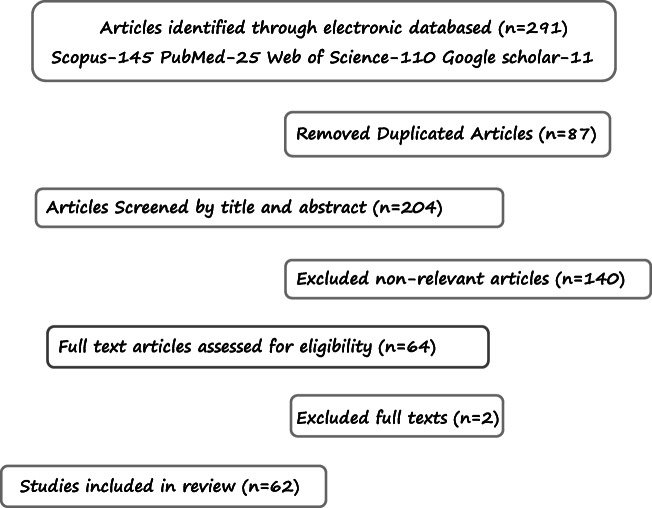
Diagram for selection of studies for the systematic review

A systematic literature survey up to August 2020 was conducted in the following databases: Scopus, PubMed, Web of Science, and Google Scholar (Fig. 2). The following inclusion criteria were employed: studies reporting significant information about the impact of TiO_2_ nanoparticles on the intestinal microbiota, available in the English language. Articles that did not meet the criteria were excluded. Classical and the newest papers were selected preferentially. The literature search entailed in the separate and joint use of a combination or keywords: “bacteria”, microbiota, TiO_2_ NPs, “impact of TiO_2_ on bacteria”, “impact of TiO2 on microbiota”, “interactions between TiO_2_ NPs and microbiota”. The literature included the following categories of papers: experimental studies and reviews. The obtained literature was manually reviewed, and the cited references were analyzed to identify the relevant studies. The search conducted at the highest sensitivity yielded 291 papers from external databases, which were subsequently collected. Next, after reviewing the titles and synopses, papers not related to the subject matter criteria were excluded, and the remaining texts were analyzed in depth to select the most relevant publications. Eventually, after identifying related papers and studies employing adequate research strategies, a total of 62 articles were analyzed.

### Causes and Consequences of Intestinal Microbiota Alterations Due to TiO_2_ NPs Exposure

**Fig. 3:**
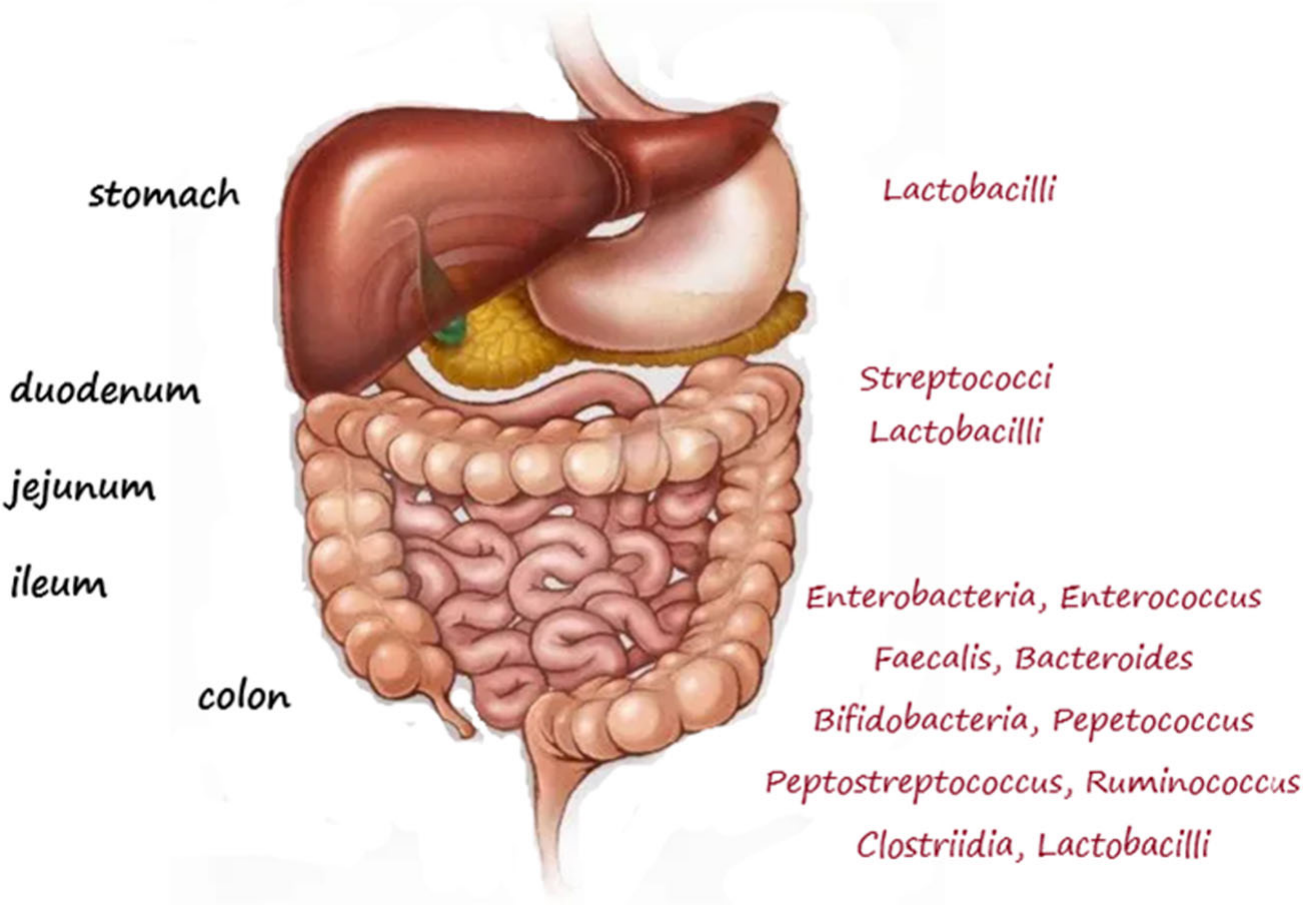
Microbiota population in different organs of the gastrointestinal (GI) tract. Based on Riasat et al. [40]

The physiological environment has a considerable significance to the interaction between inorganic nanoparticles and microorganisms [36]. Microorganism colonies can only prosper under specific microenvironmental conditions (e.g. pH, oxygen concentration, symbiotic proximity, nutrient availability) [39]. In the gastrointestinal tract, the environment is shaped by the presence of enzymes, bile, and regions with distinct pH, all of which influence the stability as well as aggregation (and size) of inorganic nanoparticles [29]. The mucous barrier, transit time, and unpredictable peristalsis will condition the transport of food, medicines, as well as the ways in which NPs may potentially interact with our alimentary tract and the microbiota present therein [39] (Fig. 3) [40]. Increased consumption of TiO_2_ NPs can have a negative impact on the human microbiome in the process of direct food consumption and/or during its passage through the intestine. Commensal bacteria and in-transit bacteria carried with the food can come into contact with TiO_2_ NPs, which can influence the resident microbiota, and consequently the host’s health [16, 41]. This may lead to inhibition of the growth and activity of gastrointestinal bacteria, in particular of the probiotic type [2]. Microbiota changes can lead to specific health problems including obesity, inflammatory bowel disease, diabetes, and rheumatoid arthritis [36, 42, 43].

Exposure to nanoparticles can take place while consuming food (it is used as pigment, filler, preservative), via the respiratory system or skin [27, 37]. In the gastrointestinal tract, nanoparticles are first acidized in the stomach, which increases their toxicity due to ion release [37]. In the small intestine, they come in contact with a variety of compounds: proteins and peptides—which can interact with the NPs forming agglomerates as well as changing their charge [44].

There have been reports on the adverse effects of E171 against intestinal epithelial bacteria in vitro [41, 45]. Agans et al. [27] did not exclude potential changes to human intestines following exposure to TiO_2_ NPs as the combination of agglomerates in cellular membranes can inhibit cells’ ability to divide or disturb the processes of absorbing nutrients. Taylor et al. [46], in a study involving 1-week in vitro exposure to TiO_2_ NPs (dosed at, respectively, 3 μg/L, 0.01 μg/L, and 0.01 g/L) observed, in the model colon, changes to multiple characteristics of bacteria phenotypes, including the production of short-chain fatty acids. Pignet et al. [47] analyzed the impact of TiO_2_ NPs (2 and 10 mg TiO_2_/kg bm/day and 50 mg TiO_2_/kg bm/day) on the large and small intestine in mice. After oral administration of TiO_2_ NPs, they reported minimum impact of NPs on the composition of gastrointestinal microbiota in mice, but at the same time observed that the same can modify the release of bacterial metabolites in vivo and influence commensal bacteria in vitro by promoting the formation of biofilm. Khan et al. [2] used TiO_2_ NPs from purified chocolate and studied its in vitro and in vivo influence on a commercial probiotic preparation typically used in the treatment of diarrhoea in children (it contained *Bacillus coagulans*, *Enterococcus faecalis*, and *Enterococcus*). The researchers demonstrated that TiO_2_ NPs obtained from chocolate inhibited the growth and activity of the probiotic preparation within the concentration range of 125–500 μg/mL in vitro. Based on the obtained results, they concluded that 20 g of the analyzed chocolate contained sufficient amounts of TiO_2_ NPs to upset the microbiological balance in the intestines of children between 2 and 8 years of age and with a stomach capacity of between 0.5 and 0.9 L; similar effects were observed in an in vivo study on white albino mice dosed at 50–100 μg/day/mouse. Pagnout et al. [48] demonstrated that the toxicity of TiO_2_ NPs is related to electrostatic interactions between bacteria (*Escherichia coli* (*E. coli*)) and nanoparticles, which lead to adsorption of the latter on the cell surface. Planchon et al. [49] corroborated the thesis on the heterogeneity of bacteria populations. In their studies, they demonstrated that after exposure to TiO_2_ NPs some bacteria were fully covered with the same, while most of the population remained free from nanoparticles, which resulted in differences in terms of proteome and metabolome. Similarly, Radziwill-Bienkowska et al. [50] observed that a part of the bacterial population remained free form TiO_2_ NPs, while another part of the same very strongly interacted with the nanoparticles. Furthermore, Waller et al. [28] demonstrated that exposure to TiO_2_ NPs caused changes to the composition of microorganisms (i.e. a shift from *Proteobacteria* to *Firmicutes phyla*) as well as lowered the colonic pH (< 5) relative to the control (> 5).

At the same time, there have been studies that revealed a limited influence of TiO_2_ NPs on human microbiota. For example, Dudefoi et al. [12] reported that TiO_2_ NPs had no significant in vitro impact on gastrointestinal microbiota. Using concentrations that simulated the one observed in an adult intestine after chewing a single piece of chewing gum (100–250 mg/L), they revealed no impact on gas production and only a negligible effect in terms of fatty acid profiles (C16: 00, C18: 00, 15: 1 w5c, 18: 1 w9c and 18: 1 w9c, *p* < 0.05) and phylogenetic composition. Agans et al. [27] demonstrated that TiO_2_ nanoparticles had limited direct influence on human gastrointestinal microbiota. After adding TiO_2_ NPs to a microorganic community, some slight reduction was observed but without changes to the overall diversity or balance thereof.

### Factors Influencing the Interaction of TiO_2_ NPs with the Microbiota, and Their Consequences

In determining the toxicity of TiO_2_ NPs, interfacial electrostatic interaction as well as physicochemical parameters of the medium (pH, ionic strength, electrolyte composition, size, temperature, light exposure) can play a rather significant role [29, 48].

### UV

TiO_2_ NPs are considered to be chemically inert without photoactivation, but they do show strong photocatalytic and antibacterial properties under UV light as they produce reactive oxygen species (ROS). Anatase is believed to be the most photocatalytically active of all titanium oxides due to its significant mobility of the electron-hole pairs and wider bandwidth range [34].

The mechanism of TiO_2_ NPs antibacterial activity under UV light has been fairly thoroughly researched [35, 51]. Planchon et al. [49] studied the proteome and metabolome of *E. coli* bacteria after exposure to TiO_2_ NPs under ultraviolet radiation and in normal light. They observed an ununiform bacterial response to the exposure from *E. coli* cells. A part of the population was able to adapt to the stress and survive for a time; the other part gradually died. The authors believe that some protein and metabolites may be used as biomarker of particle stress, e.g. chaperonin 1 and isocitrate dehydrogenase, as their content was respectively decreased and increased significantly in the presence of TiO_2_ NPs. Joost et al. [52] demonstrated in their study on living bacteria cells (*E. coli*) that exposure to TiO_2_ NPs resulted in enlargement of the cells, deformation of their membranes, and possible cytoplasm leakage after 10 min of exposure. The complete inactivation of the bacteria in thin TiO_2_ NPs layers took place after 20 min UV-A irradiation. The researchers also studied saturated and unsaturated fatty acids present in bacterial plasma membranes, which disintegrated within 10 min of exposure on photoactivated thin layers of TiO_2_ NPs. Priyadarshini et al. [53] demonstrated the inhibitive effects of TiO_2_ NPs in darkness, and enhanced effects under UV light (365 nm), on Gram-positive and Gram-negative bacteria (*Staphylococcus aureus* (*S. aureus*), *Bacillus subtilis* (*B. subtilis*), and *E. coli*). The significant bactericidal activity observed already for the minimum TiO_2_ NPs concentration (dosed at 0.5 mg/mL), and the enhancement thereof after photo-stimulation was explained by the loss of membrane integrity and increased oxidative stress on the surface of bacteria.

Some researchers have reported moderate toxicity of TiO_2_ NPs towards bacteria, even in the absence of UV radiation [54]. Dark incubation of Gram-negative *E. coli* and Gram-positive *B. subtilis* bacteria with TiO_2_ nanoparticles reduced the CFU (colony-forming units) index by, respectively, 25% and 30% [55]. Also in other studies [56], it was shown that dark incubation of *E. coli* cells with TiO_2_ NPs reduced the respective CFU by approximately four orders of magnitude in acidic pH. Zhukova et al. [57] demonstrated that 60-min exposure of *E. coli* to TiO_2_ NPs (concentrated at 0.02–0.2 mg/mL) resulted in a decrease in cell viability from 10^8^ to 10^4^ CFU/mL (colony-forming unit) in the absence of UV radiation. Qiu et al. [39] demonstrated that TiO_2_ nanoparticles (10, 50, and 100 nm in size) can inhibit the growth of commensal in vitro (*Lactobacilli*, *Enterobacteria* and *Acetobacter*) with no access to light. Radziwill-Bienkowska et al. [50] studied the interactions, under conditions with no UV radiation, between TiO_2_ NPs (food grade E171 and TiO_2_—P25) and gastrointestinal microbiota bacteria (e.g. *E. coli*) as well as those swallowed with food (e.g. *Lactococcus lactis* (*L. lactis*)). They demonstrated that bacterial growth was inhibited by TiO_2_ NPs in all the tested bacterial strains (*E. coli*, *L. lactis*, *Lactobacillus rhamnosus*, *Lactobacillus sakei*, and *Streptococcus thermophilus*), particularly by the food grade TiO_2_ NPs. They further observed that E171 may be retained in the intestine by commensal as well as in-transit bacteria carried in food. As a result, physiological changes may occur in more susceptible species.

### pH

Changes in pH significantly impact the surface charge, size, and aggregation speed of NP. Studies indicate that aggregation and stability of food grade and industrial grade TiO_2_ NPs is susceptible to solution pH in terms of particle IEP (isoelectric points) [58, 59], where industrial grade particles show IEP at approximately pH 6.8, while food grade particles at approximately pH 3.5 [59]. Lin et al. [60] demonstrated in their study that the toxicity of TiO_2_ NPs tends to decrease with growing pH. The antibacterial activity of TiO_2_ NPs (25 nm, P25) against *E. coli* was stronger at pH 5.5 than at 7.0 or 9.5. Pagnout et al. [48] observed that the viability of *E. coli* cells was significantly lowered at pH 5.5 compared to pH 7.0 or pH 9.5. Waller et al. [28] studied, during a 5-day experiment, the impact of exposure to TiO_2_ NPs (food and industrial grade) on various bacteria groups from *Proteobacteria* to *Firmicutes phyla*. They demonstrated that TiO_2_ NPs had only a slight impact on microbiological stability. They also observed that in both cases, exposure to TiO_2_ NPs resulted in decreased values of pH in the colon (< 5) compared to the control (> 5), with the exposure to food grade TiO_2_ nanoparticles inducing the highest reduction (~ pH 4) [28].

### Size

It is suspected that the size of the nano-fraction also influences disorders of gastrointestinal homeostasis as well as the development of intestinal microbiota dysbiosis [59]. Lin et al. [60] studied the toxicity of five types of TiO_2_ nanoparticles of different sizes (anatase TiO_2_ NPs with particles sizes of 10, 25, and 50 nm; rutile TiO_2_ NPs—50 nm; and mixed anatase and rutile TiO_2_ NPs—25 nm in length). The concentration of anatase TiO_2_ NPs was observed to increase, particularly for smaller particles, on the surface of *Escherichia coli* cells. It was also reported that compared to rutile NPs, anatase TiO_2_ NPs forms were more likely to bind with cell surfaces. Xiong et al. [61] demonstrated that smaller TiO_2_ NPs after UV–Vis activation of a larger surface area had a tendency to produce higher cytotoxicity. The same could be caused by generation of ROS and adsorption of bioparticles, as observed by the authors in whose study, both under biotic and abiotic conditions; ROS production was observed to increase in smaller particles. Ederm et al. [62] demonstrated higher microbiological toxicity for particles under 40 nm. In their study, the highest toxicity was reported for TiO_2_ NPs of 16.2 nm and 21.4 nm in size, which caused growth inhibition by 80% (*E. coli*) and 65% (*B. subtilis*) in the absence of light. Under light exposure, TiO_2_ nanoparticles of the same two sizes also proved to have the highest antibacterial potential.

## Conclusion

The use of titanium dioxide nanoparticles continues to give rise to controversy around the world and is subject to extensive study regarding their impact on the alimentary tract and its functioning. Currently available reports provide contradictory evidence in terms of the impact of inorganic nanoparticles on our microbiota due to the application of varying experimental models and frameworks. Advanced in vivo models need to be developed in experimental conditions to allow a more systematic study necessary for a better understanding of the variations in toxicity observed between NPs and the human microbiota.

### Future Perspective

The review discusses the impact of TiO_2_ nanoparticles on only a small group of selected bacterial strains. This was a deliberate decision that allowed me to focus on the strains directly related to my currently ongoing studies (research project - MINIATURE 3 grant (2019/03/X/NZ9/01032), “Influence of TiO_2_ nanoparticles on selected lactic and pathogenic bacterial strains, living in the human large intestine”). I aim to study the impact of TiO_2_ nanoparticles on a dozen or so selected lactic and pathogenic bacterial strains living in the human large intestine. In the study, I also employ an in vitro model of the alimentary tract to determine how the presence of TIO_2_ NPs influences the growth of the respective bacteria. This is to allow me to determine the risks related to the presence of those nanoparticles in food. The results detailing the impact of TiO_2_ NPs on the respective strains will be presented in the subsequent papers scheduled for publication next year. In the future, I intend to extend the scope of the in vitro studies using bacterial strains obtained from the intestine (Caco-2/HT29-MTX). It is my considered belief that this line of research may contribute to the minimization or even elimination of the side effects related to the use of TIO_2_ nanoparticles.
